# The Potential Benefits and Adverse Effects of Phytic Acid Supplement in Streptozotocin-Induced Diabetic Rats

**DOI:** 10.1155/2013/172494

**Published:** 2013-12-22

**Authors:** F. O. Omoruyi, A. Budiaman, Y. Eng, F. E. Olumese, J. L. Hoesel, A. Ejilemele, A. O. Okorodudu

**Affiliations:** ^1^Department of Life Sciences, Texas A&M University-Corpus Christi, Center for the Sciences, 130B, 6300 Ocean Drive, Unit 5802, Corpus Christi, TX 78412, USA; ^2^Department of Medical Biochemistry, University of Benin, Benin City, Nigeria; ^3^Department of Pathology, Clinical Chemistry Division, University of Texas Medical Branch, Galveston, TX, USA

## Abstract

In this study, the effect of phytic acid supplement on streptozotocin-induced diabetic rats was investigated. Diabetic rats were fed rodent chow with or without phytic acid supplementation for thirty days. Blood and organ samples were collected for assays. The average food intake was the highest and the body weight gain was the lowest in the group fed phytic acid supplement compared to the diabetic and normal control groups. There was a downward trend in intestinal amylase activity in the group fed phytic acid supplement compared to the other groups. The spike in random blood glucose was the lowest in the same group. We noted reduced serum triglycerides and increased total cholesterol and HDL cholesterol levels in the group fed phytic acid supplement. Serum alkaline phosphatase and alanine amino transferase activities were significantly (*P* < 0.05) increased by phytic acid supplementation. Systemic IL-1**β** level was significantly (*P* < 0.05) elevated in the diabetic control and supplement treated groups. The liver lipogenic enzyme activities were not significantly altered among the groups. These results suggest that phytic acid supplementation may be beneficial in the management of diabetes mellitus. The observed adverse effect on the liver may be due to the combined effect of streptozotocin-induced diabetes and phytic acid supplementation.

## 1. Introduction

Phytic acid (IP6) is the primary storage form of phosphorus in seeds and in some cases; it is thought to be responsible for their remarkable longevity of up to 400 years [1, 2]. Phytic acid is also found in significant quantities in roots and tubers. However, with the exception of avocado fruit, the levels of phytic acid in fruits are generally lower than the levels in seeds. Phytic acid is generally considered as an antinutrient because of its strong ability to chelate multivalent metal ions, precipitate, and decrease the availability of these minerals as a result of the formation of very insoluble salts that are poorly absorbed from the gut [3, 4]. The involvement of phosphorylated inositol (IP1 to IP6), especially IP6 in insulin secretion, has been reported [5]. For example, an extract from the skin of sweet potato has been shown to aid in the control of type II diabetes by decreasing insulin resistance in the affected patients [6]. Of note, however, is the increase intracellular concentration of D-myoinositol 1,2,3,4,5,6-hexakisphosphate (IP6) in cells that are involved in insulin secretion and several other cell types [7–9]. It is believed that phosphorylated inositol, especially phytic acid have roles in the secretion of insulin by the beta cells of pancreas. Phytic acid has also been suggested to inhibit the initiation of plaque formation [5] and lower serum cholesterol and triglycerides [5]. Grases et al. [10] reported that phytic acid supplementation reduces age related aortic calcification which suggests the involvement of this compound in the protection of the arteries against hardening. However, the strong correlation between systemic inflammation with a low degree of plaque stability points to the reduction of the breaking up of plague that blocks blood flow by decreased inflammation and phytic acid has been shown to reduce inflammation and also to inhibit platelet aggregation [5, 11, 12]. It is believed that the presence of antioxidants in the pigment of red wine reduces the prevalence of heart disease among red wine users [5] which may support the use of phytic acid, a physiological antioxidant, in the reduction of heart disease. The successful use of phytic acid as an antioxidant includes its ability to chelate iron and remove it from circulation and in the process, block the generation of iron-driven hydroxyl radical as well as the suppression of lipid peroxidation [4, 13]. It has also been suggested that phytic acid may directly bind some enzymes in the process and alter the activities of those enzymes. Phytic acid extract from the skin of sweet potato has been shown to aid in the control of Type II diabetes by decreasing insulin resistance in the affected patients. However, the mode of action of phytic acid supplementation in the management of diabetes is not clear. In this study, the potential benefits and adverse effects of phytic acid supplement consumption in streptozotocin-induced diabetic rats were evaluated.

## 2. Materials and Methods 

### 2.1. Animals

Eighteen (18) adult Sprague rats were assigned by weight into three groups for a 30-day study (6 rats per group), average body weight (202.03 ± 15.77 g). The groups were composed as follows: Healthy rats receiving normal diet (Normal); diabetic rats fed normal diet (Diabetic); and diabetic rats fed phytic acid supplement (4% of rodent chow; Phytic Acid Treated Diabetic). Two of the three groups received a single injection of streptozotocin (Sigma-Aldrich, 60 mg/kg body weight in 0.05 M-citrate buffer, pH 4.5) intraperitoneally. The third group, the normal control group, was injected intraperitoneally with an equivalent amount of buffer (0.05 M-citrate buffer, pH 4.5). Rats were housed in cages with solid flooring covered with a bedding material and were allowed to free access to food and water. The cages were cleaned daily. Approval for the study was obtained after the review of the protocol by the Institutional Animal Care and Use Committee (IACUC) of the University of Texas Medical Branch Health Research Services, Galveston, Texas, with Protocol no. 1202004. After 8 days, blood was obtained from the saphenous vein and the level of blood glucose was determined. Body weight change and total food intake were recorded weekly. Animals were euthanized with pentobarbital (120 mg/kg) as the anesthetic agent on day 30 after the commencement of the feeding trial excluding the 8 days period for the development of the animal model of the disease during which all the rats were fed control diet. Organs and blood were collected.

### 2.2. Assays

Blood glucose and lipid profile were measured using Vitros 5,1 FS, Ortho-Clinical Diagnostics Inc., USA. Serum electrolytes, protein, albumin, uric acid and creatinine levels, and alkaline phosphatase and alanine amino transferase activities were measured using reagent kits from Stanbio, USA. The levels of IL-1*β* and IL-6 in the serum were measured using ELISA kits (Thermo Scientific/Pierce Biotechnology, IL, USA) and Bio-Rad Microplate reader. Lipogenic enzyme activities in the liver were determined by measuring the change in extinction due to NADP reduction or NADH oxidation [14].

### 2.3. Statistical Analysis

Results are presented as means ± SEM. Analysis of variance (ANOVA) was used to test for differences among the groups. Post hoc analysis was carried out using Duncan's multiple range test to test for significant difference among the means (*P* < 0.05).

## 3. Results and Discussion

**Figure 1:**
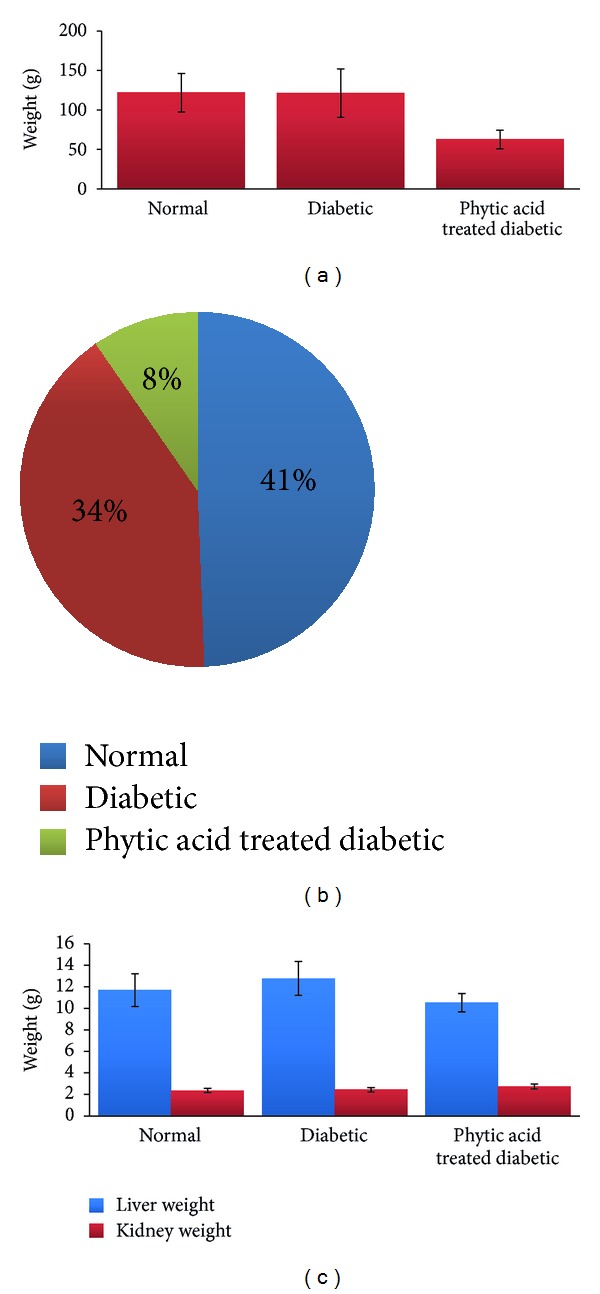
(a) Body weight changes in diabetic rats fed phytic acid supplement. Values were not significantly different among the groups (*P* > 0.05). (b) Percentage spike in random blood glucose in diabetic rats fed phytic acid supplement. (c) Organ weights in diabetic rats fed phytic acid supplement. Values were not significantly different among the groups (*P* > 0.05).

Phytic acid has been reported to be involved in insulin secretion, inhibition of the initiation of plaque formation [5], and reduction of serum lipids [4]. Dilworth et al. [15] reported the lowering of blood glucose by phytic acid supplementation which may be beneficial in the management of diabetes. However, fiber and phytic acid occur together in fiber-rich diets [16] and it is believed that phytic acid as opposed to fiber is mostly responsible for the reduced availability of minerals in animals. The 4 g/100 g weight food fed in this study simulates the level of soluble or insoluble fiber used in previous studies [17, 18]. Figures 1(a), 1(b), and 1(c) show body weight changes, percentage spike in random blood glucose, and organ weights in diabetic rats fed phytic acid supplement.

**Figure 2:**
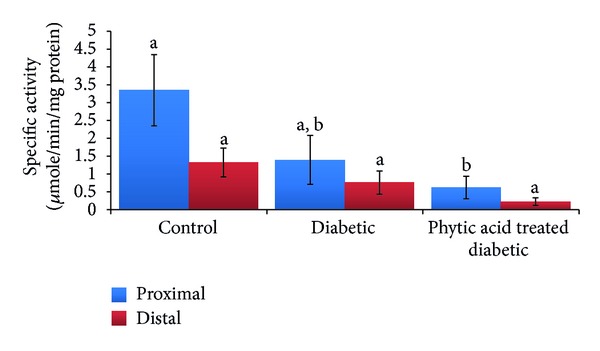
Amylase activity in the intestinal mucosa of diabetic rats fed phytic acid. Figures that share different letter superscripts are significantly different (*P* < 0.05).

The average food intake was the highest in the group fed phytic acid supplement (48 g per day) compared to the other groups (32 g for nondiabetic control group and 34 g for diabetic control group). Although the consumption of food was the highest in the diabetic rats fed phytic acid supplement, it failed to be transformed into increased weight gain. This suggests that phytic acid supplementation may interfere with food digestion/absorption. This may be beneficial to Type 2 diabetic patients. An earlier report showed that phytate extract from the skin of sweet potato may aid in the control of Type 2 diabetes by decreasing insulin resistance in the affected patients [6]. Data from our study is consistent with another earlier report [19] which showed a significant depression of growth in rats fed sodium phytate but with no changes in the food intake. The difference in food intake between the studies may be due to the type of phytate fed. While Onomi et al. [19] used sodium phytate supplement in their study, calcium-magnesium phytate (the naturally occurring form) supplement was used in our study. Several systemic peptides have been shown to regulate appetite and body weight. For example, plasma leptin levels have been shown to decrease rapidly during fasting and increase postprandially [20]. Adiponectin level on the other hand increases during fasting and leads to an increase in food intake. It also has important roles in energy homeostasis, regulation of insulin sensitivity, and protection against vascular injury and atherosclerosis. Reduced adiponectin levels have been reported to be associated with increased production of proinflammatory proteins (IL-6 and C-reactive protein) and strongly correlated with coronary artery disease [21, 22]. The stomach derived peptide ghrelin regulates food intake and body weight, and the level of ghrelin is believed to rise before meals and fall after meals [23]. Gastric ghrelin expression and peptide secretion are increased by food deprivation and normalize during refeeding [24]. To elucidate the probable mode of action of phytic acid supplement on food consumption and body weight, further studies are needed to establish the potential role of phytic acid derivatives on these vital systemic peptides involved in the regulation of appetite and body weight. The diabetic group showed an increase in the liver weight while the phytic acid supplemented group showed a decrease in the liver weight although these changes were not significant statistically (*P* > 0.05). Diabetes is associated with a wide spectrum of liver conditions including nonalcoholic fatty liver disease and cirrhosis. The elevated liver weight seen in the diabetic rats may be indicative of the presence of liver disease which is ameliorated by phytic acid supplementation. The percentage spike in random blood glucose was the lowest in the group fed phytic acid supplement (8%) and the highest in the nondiabetic control group (41%). This could be due to the observed downward trend in intestinal amylase activity (Figure 2) in the group fed phytic acid supplement compared to the other groups. Earlier study by Thompson [25] showed impairment of starch digestion by phytic acid supplementation. We noted a significant reduction (*P* < 0.05) in intestinal amylase activity in the group fed phytic acid supplement and a downward trend in the diabetic group compared to the nondiabetic control group. Streptozotocin has been proposed to cause a reduction in amylase activity by interfering with calcium and magnesium homeostasis and amylase gene expression [26]. Phytic acid on the other hand inhibits *α*-amylase [27], thereby leading to a further reduction in the measured activity as observed in our study. The antidiabetic function of phytic acid supplement may be through the decrease in the activity of intestinal amylase which is indicative of lesser products of carbohydrate digestion formation and subsequently absorption, leading to a decreased percentage spike in random blood glucose [17].

**Figure 3:**
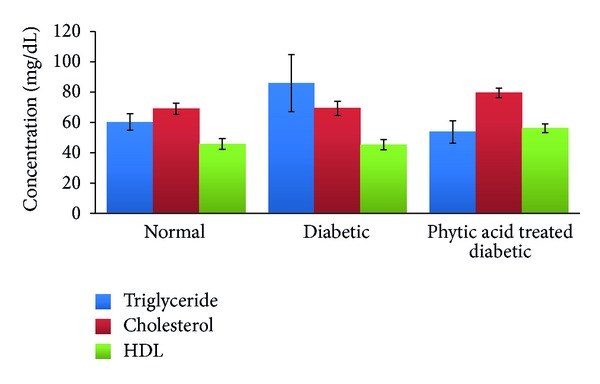
Lipid profile in diabetic rats fed phytic acid supplement. Values were not significantly different among the groups (*P* > 0.05).

We noted an increase in the serum triglycerides and total cholesterol levels in the diabetic control rats but reduced serum triglycerides and increased total cholesterol and HDL cholesterol levels in the group fed the phytic acid supplement (Figure 3). These differences were however not significantly different statistically. The changes seen in the diabetic control rats were expected changes. Phytic acid has been shown to reduce lipase activity, total cholesterol, low density lipoprotein, hepatic total lipids, and hepatic triglycerides while it increases high density lipoprotein levels [28, 29]. The elevated cholesterol noted in our study may be attributed to the use of calcium-magnesium phytate rather than sodium phytate used in other studies. In an in vitro study carried out by Yuangklang et al. [30], calcium phytate complex was shown not to bind bile acids. They also suggested that this may reduce fecal bile excretion and increase serum cholesterol in vivo. Reduced triglyceride and increased HDL cholesterol levels are desirable in the management of diabetes. However, the observed increase in serum total cholesterol may be a drawback in the use of phytic acid supplement in the management of diabetes. Of note, however, is the association between decreased body weight and increased HDL level in the serum of rats fed phytic acid supplement, indicating that decrease in body weight due to phytic acid supplementation may be sensitive to elevated serum HDL cholesterol. However, the evaluation of the entire lipid profile which includes LDL, VLDL, and lipid moieties is required to ascertain whether the tissues are taking up the excess lipids or they are improving the fuel utilization. The observed increase in systemic cholesterol may also be due to increased 3-hydroxy-3-methylglutaryl coenzyme A (HMG-CoA) reductase activity. There are reports [31, 32] indicating increased HMG-CoA reductase activity in diabetic rats. A deficiency in insulin has been shown to be associated with an increase in cholesterol levels due to the enhanced mobilization of lipids from adipose tissue to the plasma [33]. HMG-CoA reductase inhibitors are widely used for the treatment of hypercholesterolemia since inhibitors of this enzyme activity block the conversion of HMG-CoA to mevalonate, the rate limiting step in the cholesterol biosynthetic pathway. We suggest further study to establish whether the observed increase in systemic cholesterol level is due to phytic acid derivatives effect on HMG CoA reductase activity in the liver and adipose tissues.

In this study, we also tested the effect of phytic acid supplementation on serum content of calcium and magnesium and alanine amino transferase and alkaline phosphatase activities (Table 1). There was no significant change (*P* > 0.05) in the levels of these minerals in the serum. This may be due to compensatory mechanisms such as resorption of stored calcium and magnesium from bone, muscle, liver, and digestive tract resulting in the almost unaltered level of these minerals in the serum in spite of their reduced absorption [28, 29]. These compensatory responses are supported by the observed significant increase (*P* < 0.05) in serum alkaline phosphatase activity in the group fed phytic acid supplement. The effect of long-term consumption of phytic acid supplement on these compensatory responses in diabetes needs further investigation.

**Figure 4:**
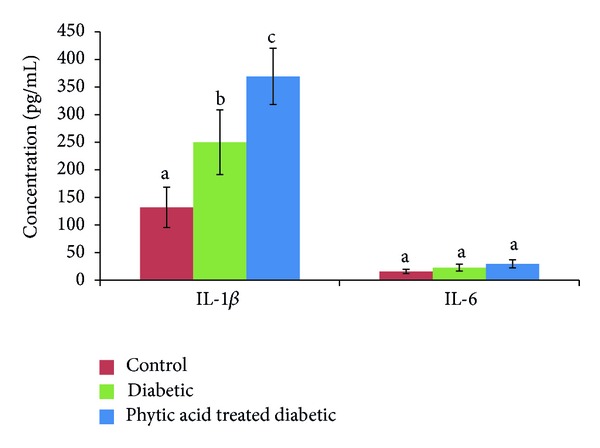
Systemic IL-1*β* and IL-6 levels in rats fed phytic acid supplement. Figures that share different letter superscripts are significantly different (*P* < 0.05).

**Figure 5:**
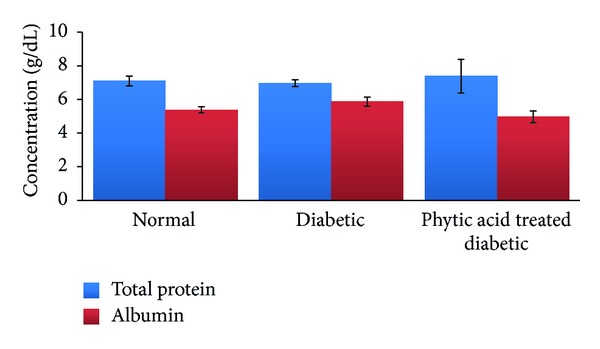
Total protein and albumin levels in the blood of diabetic rats fed phytic acid supplement. Values were not significantly different among the groups (*P* > 0.05).

**Figure 6:**
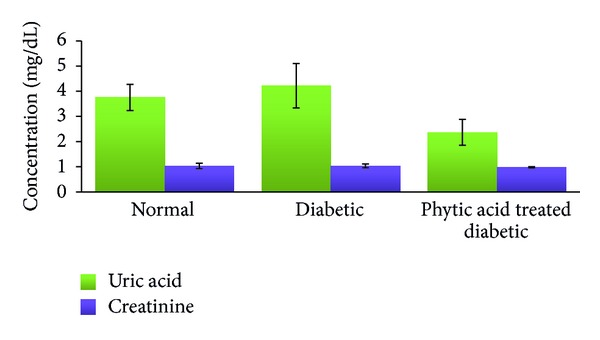
Uric acid and creatinine levels in diabetic rats fed phytic acid supplement. Values were not significantly different among the groups (*P* > 0.05).

Alanine amino transferase is a cellular enzyme that is present in low concentration in serum under normal conditions. Significant elevation in serum concentration results from either increased synthesis of this enzyme or increased leakage from damaged cells [34]. Changes in liver enzyme activity are often detectable before physical symptoms of tissue damage are apparent. We noted significant increase (*P* < 0.05) in the activity of serum alanine amino transferase in the group fed phytic acid supplement compared to the nondiabetic control group. This significant elevation in serum alanine amino transferase activity may be indicative of liver damage due to the combined effect of streptozotocin-induced diabetes and 4% level supplementation of phytic acid. Our data on IL-1*β* and IL-6 (Figure 4) support this observation as we noted significant increase in IL-1*β* level in the diabetic control group compared to the normal control group. Further significant elevation in the serum level of this cytokine was seen in the group fed phytic acid supplement. Serum IL-6 levels were not significantly altered among the groups. We however noted increasing trend in the diabetic control and the treatment groups compared to the nondiabetic control group. The significant elevation of serum IL-1*β* (a proinflammatory cytokine) level in the group fed phytic acid supplement may have resulted from hypertrophied liver tissue with subsequent leakage of alanine amino transferase and alkaline phosphatase from the liver into the serum. Systemic IL-1*β*, TNF-*α*, and IL-6 levels have been found to be elevated during aging and diabetes [35]. Recent report [36] showed that the levels of IL-1*β*, IL-10, IL-12, IL-13, and TNF-*α* preceded the elevation of serum liver enzymes. We hypothesize that the increase in serum alanine amino transferase and alkaline phosphatase activities may be due to liver damage resulting from the combined effect of streptozotocin-induced diabetes and phytic acid supplementation. However, to better understand whether the observed effects on the liver are due to streptozotocin alone, we suggest further study on these parameters on day zero and day eight when diabetes was confirmed. We observed downward trend in albumin level (Figure 5) in the diabetic group fed phytic acid supplement compared to the diabetic control group which may be indicative of early stage of liver dysfunction. In this short-term study, there were no significant changes in serum creatinine and uric acid levels among the groups (Figure 6). This is indicative of no adverse effect of phytic acid supplementation on renal function. However, the observed downward trend in serum uric acid level in diabetic rats fed phytic acid supplement compared to the other groups may be indicative of lower risk of gout development.

**Figure 7:**
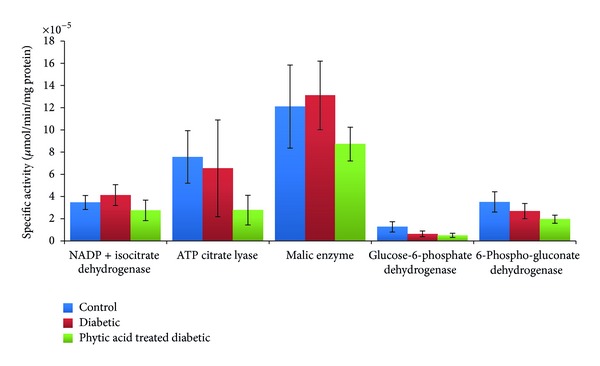
Metabolic enzymes activities in the liver of diabetic rats fed phytic acid supplement. Values were not significantly different among the groups (*P* > 0.05).

Key reactions of pentose phosphate pathway are catalyzed by glucose-6-phosphate dehydrogenase and 6-phosphogluconate dehydrogenase. These reactions generate NADPH for the synthesis of steroids and fatty acids. Malic enzyme similarly generates NADPH for reductive biosynthesis and ATP citrate lyase produces acetyl CoA for the synthesis of steroids and fatty acids. In this study, lipogenic enzymes were not significantly altered among the groups. However, we noted a downward trend in liver lipogenic enzyme activities in diabetic rats fed phytic acid supplement. Onomi et al. [19] reported depression in hepatic glucose-6-phosphate dehydrogenase and malic enzyme activities by dietary phytate supplementation in rats fed a high-sucrose diet. They reported a noncorrelation of hepatic lipogenic enzyme activity with serum concentrations of lipids. They concluded that serum lipids may not be sensitive to phytic acid action. Their suggestion that dietary phytate may be protective against fatty liver by a mechanism involving depressed lipogenesis without affecting serum lipids is consistent with the findings in this study involving diabetic rats (Figure 7).

The observed decrease in hepatic lipogenic enzyme activities in this study may also be explained by Yuangklang et al. [30] report that calcium-phytate complex does not bind bile acids which suggests maintenance of bile acids pool and nonutilization of cholesterol for bile acid synthesis with subsequent elevation in serum cholesterol.

## 4. Conclusions

The reduced intestinal amylase activity is indicative of reduced products of carbohydrate digestion available for absorption and this may account for the observed lowest random blood glucose spike in the group fed phytic acid supplement. Data from this study also showed reduced triglyceride and body weight gain and increased HDL cholesterol levels in the blood, which are beneficial in the management of diabetes. The observed adverse effect on the liver may be due to the combined effect of streptozotocin-induced diabetes and phytic acid supplementation. Overall, our data was in rat models which could only provide an indirect evaluation of the supplement for human use.

## Figures and Tables

**Table 1:** Serum calcium and magnesium levels and alanine amino transferase and alkaline phosphatase activities in diabetic rats fed phytic acid supplement.

	Normal	Diabetic	Phytic acid treated diabetic
Calcium (mg/dL)	6.87 ± 0.53	6.33 ± 0.65	6.90 ± 0.33
Magnesium (mEq/L)	3.05 ± 0.13	2.72 ± 0.08	2.82 ± 0.14
Alkaline phosphatase (U/L)	94.67 ± 14.98^a^	125.10 ± 24.42^a^	231.1 ± 35.06^b^
Alanine aminotransferase (U/L)	27.23 ± 2.15^a^	30.90 ± 2.83^a,b^	45.67 ± 6.40^b^

Values in rows that share different letter superscripts are significantly different (*P* < 0.05).

## References

[1] Owen RW, Weisgerber UM, Spiegelhalder B, Bartsch H (1996). Faecal phytic acid and its relation to other putative markers of risk for colorectal cancer. *Gut*.

[2] Lopez HW, Coudray C, Bellanger J, Younes H, Demigné C, Rémésy C (1998). Intestinal fermentation lessens the inhibitory effects of phytic acid on mineral utilization in rats. *Journal of Nutrition*.

[3] Phillippy BQ (2003). Inositol phosphates in foods. *Advances in Food and Nutrition Research*.

[4] Zhou JR, Erdman JW (1995). Phytic acid in health and disease. *Critical Reviews in Food Science and Nutrition*.

[5] Vanderlinden ND, Vucenik I (2004). *Too Good to Be True?*.

[6] Ludvik B, Waldhäusl W, Prager R, Kautzky-Willer A, Pacini G (2003). Mode of action of *Ipomoea batatas* (Caiapo) in type 2 diabetic patients. *Metabolism*.

[7] French PJ, Bunce CM, Stephens LR (1991). Changes in the levels of inositol lipids and phosphates during the differentiation of HL60 promyelocytic cells towards neutrophils or monocytes. *Proceedings of the Royal Society B*.

[8] Jackson TR, Hallam TJ, Downes CP, Hanley MR (1987). Receptor coupled events in bradykinin action: rapid production of inositol phosphates and regulation of cytosolic free Ca^2+^ in a neural cell line. *The EMBO Journal*.

[9] Li G, Pralong WF, Pittet D, Mayr GW, Schlegel W, Wollheim CB (1992). Inositol tetrakisphosphate isomers and elevation of cytosolic Ca^2+^ in vasopressin-stimulated insulin-secreting RINm5F cells. *Journal of Biological Chemistry*.

[10] Grases F, Sanchis P, Perello J (2008). Phytate reduces age-related cardiovascular calcification. *Frontiers in Bioscience*.

[11] Sudheer MK, Sridhar BR, Babu SK, Bhilegaonkar PM, Shirwaikar A, Unnikrishnan MK (2004). Antiinflammatory and antiulcer activities of phytic acid in rats. *Indian Journal of Experimental Biology*.

[12] Kamp DW, Israbian VA, Yeldandi AV, Panos RJ, Graceffa P, Weitzman SA (1995). Phytic acid, an iron chelator, attenuates pulmonary inflammation and fibrosis in rats after intratracheal instillation of asbestos. *Toxicologic Pathology*.

[13] Minihane AM, Rimbach G (2002). Iron absorption and the iron binding and anti-oxidant properties of phytic acid. *International Journal of Food Science and Technology*.

[14] Storey JM, Bailey E (1978). Effect of streptozotocin diabetes and insulin administration on some liver enzyme activities in the post-weaning rat. *Enzyme*.

[15] Dilworth LL, Omoruyi FO, Simon OR, Morrison EYSA, Asemota HN (2005). The effect of phytic acid on the levels of blood glucose and some enzymes of carbohydrate and lipid metabolism. *West Indian Medical Journal*.

[16] Torre M, Rodriguez AR, Saura-Calixto F (1991). Effects of dietary fiber and phytic acid on mineral availability. *Critical Reviews in Food Science and Nutrition*.

[17] Omoruyi F, Adamson I (1993). Digestive and hepatic enzymes in streptozotocin-induced diabetic rats fed supplements of dikanut (*Irvingia gabonensis*) and cellulose. *Annals of Nutrition and Metabolism*.

[18] Omoruyi F, Adamson I (1994). Effect of supplements of dikanut (*Irvingia gabonensis*) and cellulose on plasma lipids and composition of hepatic phospholipids in streptozotocin- induced diabetic rat. *Nutrition Research*.

[19] Onomi S, Okazaki Y, Katayama T (2004). Effect of dietary level of phytic acid on hepatic and serum lipid status in rats fed a high-sucrose diet. *Bioscience, Biotechnology and Biochemistry*.

[20] McGregor GP, Desaga JF, Ehlenz K (1996). Radioimmunological measurement of leptin in plasma of obese and diabetic human subjects. *Endocrinology*.

[21] Engeli S, Feldpausch M, Gorzelniak K (2003). Association between adiponectin and mediators of inflammation in obese women. *Diabetes*.

[22] Zoccali C, Mallamaci F, Tripepi G (2002). Adiponectin, metabolic risk factors, and cardiovascular events among patients with end-stage renal disease. *Journal of the American Society of Nephrology*.

[23] Wiedmer P, Nogueiras R, Broglio F, D’Alessio D, Tschöp MH (2007). Ghrelin, obesity and diabetes. *Nature Clinical Practice Endocrinology and Metabolism*.

[24] Cummings DE, Purnell JQ, Frayo RS, Schmidova K, Wisse BE, Weigle DS (2001). A preprandial rise in plasma ghrelin levels suggests a role in meal initiation in humans. *Diabetes*.

[25] Thompson LU (1988). Antinutrients and blood glucose. *Food Technology*.

[26] Patel R, Yago MD, Victoria EM, Shervington A, Singh J (2004). Mechanism of exocrine pancreatic insufficiency in streptozotocin-induced diabetes mellitus in rat: effect of cholecystokinin-octapeptide. *Molecular and Cellular Biochemistry*.

[27] Kuppusamy A, Muthusamy U, Andichetiar Thirumalaisamy S, Varadharajan S, Ramasamy K, Ramanathan S (2011). In vitro (*α*-glucosidase and *α*-amylase inhibition) and in vivo antidiabetic property of phytic acid (IP6) in streptozotocin-nicotinamide- induced type 2 diabetes mellitus (NIDDM) in rats. *Journal of Complementary and Integrative Medicine*.

[28] Lee S-H, Park H-J, Cho S-Y (2005). Effects of dietary phytic acid on serum and hepatic lipid levels in diabetic KK mice. *Nutrition Research*.

[29] Liu N, Ru Y, Wang J, Xu T (2010). Effect of dietary sodium phytate and microbial phytase on the lipase activity and lipid metabolism of broiler chickens. *British Journal of Nutrition*.

[30] Yuangklang C, Wensing T, Lemmens AG, Jittakhot S, Beynen AC (2005). Effect of sodium phytate supplementation on fat digestion and cholesterol metabolism in female rats. *Journal of Animal Physiology and Animal Nutrition*.

[31] Prince PSM, Kannan NK (2006). Protective effect of rutin on lipids, lipoproteins, lipid metabolizing enzymes and glycoproteins in streptozotocin-induced diabetic rats. *Journal of Pharmacy and Pharmacology*.

[32] Rydgren T, Sandler S (2009). The protective effect of simvastatin against low dose streptozotocin induced type 1 diabetes in mice is independent of inhibition of HMG-CoA reductase. *Biochemical and Biophysical Research Communications*.

[33] Karthikesan K, Pari L, Menon VP (2010). Antihyperlipidemic effect of chlorogenic acid and tetrahydrocurcumin in rats subjected to diabetogenic agents. *Chemico-Biological Interactions*.

[34] Pincus MR, Sehaffner JR (1996). *Assessment of Liver Function in Clinical Diagnosis and Management By Laboratory Methods*.

[35] Roeske-Nielsen A, Fredman P, Mansson JE, Bendtzen K, Buschard K (2004). Beta-galactosylceramide increases and sulfatide decreases cytokine and chemokine production in whole blood cells. *Immunology Letters*.

[36] Kakisaka K, Takikawa Y (2013). Elevation of serum cytokines preceding elevation of liver enzymes in a case of drug-induced liver injury. *Hepatology Research*.

